# Comparison of the Antimicrobial Activities of Four Honeys From Three Countries (New Zealand, Cuba, and Kenya)

**DOI:** 10.3389/fmicb.2018.01378

**Published:** 2018-06-25

**Authors:** Gianluca Morroni, José M. Alvarez-Suarez, Andrea Brenciani, Serena Simoni, Simona Fioriti, Armanda Pugnaloni, Francesca Giampieri, Luca Mazzoni, Massimiliano Gasparrini, Emanuela Marini, Marina Mingoia, Maurizio Battino, Eleonora Giovanetti

**Affiliations:** ^1^Dipartimento di Scienze Biomediche e Sanità Pubblica, Università Politecnica delle Marche, Ancona, Italy; ^2^Escuela de Medicina Veterinaria y Zootecnia, Facultad de Ciencias de la Salud, Grupo de Investigación en Biotecnología Aplicada a Biomedicina (BIOMED), Universidad de Las Américas, Quito, Ecuador; ^3^Dipartimento di Scienze Cliniche Specialistiche ed Odontostomatologiche (DISCO), Università Politecnica delle Marche, Ancona, Italy; ^4^Dipartimento di Scienze Cliniche e Molecolari, Università Politecnica delle Marche, Ancona, Italy; ^5^Dipartimento di Scienze della Vita e dell’Ambiente, Università Politecnica delle Marche, Ancona, Italy

**Keywords:** honey, antimicrobial activity, minimum active dilution, biofilm, bacterial ultrastructural analysis

## Abstract

Skin and chronic wound infections are an increasing and urgent health problem worldwide. Their management is difficult and the development of antibiotic resistance by both planktonic and biofilm-associated bacteria necessitates the use of alternative treatments. The purpose of this study was to compare the antimicrobial activity of four honeys from different floral and geographical origins: *Melipona beecheii* honey (Cuba) and three *Apis mellifera* honeys [Manuka honey (New Zealand), *A. mellifera* honey (Cuba), and African honey (Kenya)]. The physicochemical parameters were within the ranges reported for these honeys and *M. beecheii* honey stood out due to its acidic character. An agar incorporation technique was used to determine the minimum active dilution of each honey against 52 clinical isolates (34 Gram-positive, 17 Gram-negative, and 1 *Candida albicans*). The antibiofilm activity of honeys was tested by assessing their ability to inhibit biofilm formation and to disrupt preformed biofilms. Overall, *M. beecheii* honey had the highest antimicrobial and antibiofilm activity, although a marked disruption in preformed biofilms was shared by all tested honeys. Structural changes induced by *M. beecheii* honey on *Staphylococcus aureus* and *Pseudomonas aeruginosa* cells were observed by transmission electron microscopy suggesting that this honey has a potent antimicrobial action and may be an excellent candidate for the development of topical preparations for the treatment of infected wounds.

## Introduction

The development of new classes of antibiotics has diminished over the past 20 years, with few companies remaining active in this pharmaceutical area. At the same time, antibiotic-resistant bacteria have significantly increased, largely due to overuse and misuse of antibiotics. This antibiotic crisis is in progress globally and involves drugs not only for systemic use, but also for topical use: for instance, the management of chronic wounds often requires long-term therapy (e.g., diabetic foot, venous ulcers, and pressure ulcers). Given the difficulty in treating these infections, the antimicrobial potential of unconventional, non-antibiotic treatments has evoked interest. In particular, the potential role of honey — renowned for its wound-healing properties since ancient times — has been rediscovered in recent years (Molan, 1999; Maddocks and Jenkins, 2013; Cooper, 2016). Diverse honeys differ in the potency of their antibacterial activity depending on plant source, geographical location, and harvesting, processing, and storage conditions. Honey may kill bacteria, and this effect is largely attributed to high osmolarity, acidity (pH and free acidity), low water activity, hydrogen peroxide production, and the presence of other phytochemical components (Kwakman et al., 2010; Kwakman and Zatt, 2012; Alvarez-Suarez et al., 2018).

In particular, Manuka medical-grade honey, one of the major and most extensively investigated varieties, has potent bactericidal activity and is currently approved for clinical application in infected wound management (Kwakman and Zatt, 2012). This honey is derived from nectar collected by honeybees (*Apis mellifera*) from the Manuka bush (*Leptospermum scoparium*) indigenous to New Zealand and Australia. It contains very high concentrations of the antibacterial compound 1,2-dicarbonyl methylglyoxal (Adams et al., 2008; Mavric et al., 2008). Continuous exposure to sub-lethal concentrations of Manuka honey for 28 days failed to select for honey-resistant bacteria (Cooper et al., 2010). However, incomplete knowledge of honey’s antibacterial factors and their contribution to bacterial killing hampers general applicability of honey in clinical settings.

To the best of our knowledge, there are very few studies investigating traditional African and Cuban honeys for potential wound care (Ndip et al., 2007; Alvarez-Suarez et al., 2010b). Very recently, two Cuban polifloral honeys (one from *A. mellifera* bees and one from *Melipona beecheii* bees) were compared in our laboratory. As a result, *M. beecheii* honey turned out to be a more important source of bioactive compounds with better biological properties and also exhibited a higher antimicrobial activity (Alvarez-Suarez et al., 2018). Based on these results, the aim of this study was to investigate and compare the *in vitro* antimicrobial activities of two Cuban honeys, an African (Kenyan) honey and the widely investigated Manuka honey.

## Materials and Methods

### Honey Samples

Four different honeys, all collected between 2013 and 2015, were used. Three honeys from *A. mellifera*: (i) Manuka, from New Zealand, unifloral source (*L. scoparium*) (Alvarez-Suarez et al., 2016); (ii) polyfloral *A. mellifera* honey from Cuba and (iii) Kenyan honey, unifloral source (*Faurea saligna*), henceforth referred to as “African,” and one (iv) polyfloral honey from *M. beecheii* from Cuba (Alvarez-Suarez et al., 2018).

Honey samples (250 g) were obtained directly from beekeepers, except for Manuka honey, which was obtained from New Zealand Honey LTH, imported to Italy by Efit Srl. The samples were packed and sealed in amber glass bottles and stored at 4°C in the dark until processed. The botanical origin of honey was confirmed by traditional qualitative microscopic analysis and frequency determination of the classes of pollen grains in the samples (Louveaux et al., 1978; Von Der Ohe et al., 2004). Before analyses were performed, the samples were kept at room temperature overnight.

### Physicochemical Parameters, Chemical Composition and Total Antioxidant Capacity

To confirm the quality parameters, all the honey samples were tested using the usual physicochemical tests, such as ashes (%), color (mm Pfund), Diastase activity (Schade units), free acidity (meq/kg), pH, electrical conductivity (mS/cm), moisture (%) and the hydroxymethylfurfural (HMF) test (mg/kg) (Official Methods of Analysis of the (Association of Official Analytical Chemists [AOAC], 1990; EU Council, 2002).

For the chemical composition analysis, the Folin-Ciocalteu method (Singleton et al., 1999) was used for total phenolic content (TPC) determination and results were expressed as mg of gallic acid equivalents (GAE) per 100 g of honey (mg GAE/100 g of honey). The aluminum chloride colorimetric method was used for the total flavonoid content (TFC) analysis (Chang et al., 2002) and results were expressed as mg of (+)-catechin equivalents per 100 g of honey (mg CE/100 g of honey). Total carotenoid content (TCC) was determined spectrophotometrically (Alvarez-Suarez et al., 2010b) and results were expressed as mg of β-carotene equivalents per kg of honey (mg βcarotE/kg of honey). Free amino acids content was determined by the Cd-ninhydrin method (Doi et al., 1981) and results were expressed as mg of L-leucine equivalents per 100 g of honey (mg LE/100 g of honey), whilst the Bradford method was used for protein content (Alvarez-Suarez et al., 2010b) and results were expressed as mg of bovine serum albumin equivalents per 100 g of honey (mg BSA/100 g of honey). Vitamin C content was determined using a reversed-phase HPLC-DAD system. Ascorbic acid was used as standard and results were expressed as mg VitC/100 g of honey (Alvarez-Suarez et al., 2010a). Folic acid content was determined by RP-HPLC system following the methodology previously described for the determination of water-soluble vitamins in honey (Ciulu et al., 2011). Vitamin B9 (folic acid) was used as standard and results were expressed as mg folic acid/kg of honey.

The ferric reducing antioxidant power (FRAP) (Benzie and Strain, 1996) and DPPH radical scavenging assays (Velázquez et al., 2003) were used to determine the total antioxidant capacity (TAC) of the honey samples. Trolox was used for the calibration curves in both assays and the results were expressed as μmoles of Trolox equivalents per 100 g of honey (μmol TE/100 g of honey).

### Microbial Strains

A collection of 52 clinical isolates, recovered from human specimens in laboratories in central Italy between April 2013 and September 2015, was used. In order to achieve easier comparison, this collection (34 Gram-positive, 17 Gram-negative, and 1 *Candida albicans*) was in part the same recently used in a comparative study of the physicochemical, chemical, and biological properties of the two Cuban honeys (Alvarez-Suarez et al., 2018).

### Determination of Minimum Active Dilution of Each Honey

The minimum active dilution (MAD) was determined by an agar incorporation technique with Mueller-Hinton agar (Oxoid, Basingstoke, England), supplemented with 5% sheep blood for streptococci. A dilution series with final honey concentrations in the range of 1 — 20% (v/v), in 1% increments, was used for susceptibility assays. Duplicate control plates with no honey were included to confirm the viability of the cultures. Each culture was inoculated on agar plates using an auto-pipettor and plates were incubated at 37°C overnight. The MAD was the lowest honey dilution at which microbial growth was completely inhibited (Alvarez-Suarez et al., 2018).

### Antibiofilm Activity

Biofilm formation and quantification were assessed using previously described microtiter-plate test. The cut-off OD (OD_c_) for the microtiter-plate test was three standard deviations above the mean OD of the negative control. Strains were classified as follows: OD < OD_c_ non-adherent; OD_c_ < OD < 2 X OD_c_ weakly adherent; 2 X OD_c_ < OD < 4 X OD_c_ moderately adherent; 4 X OD_c_ < OD strongly adherent (Stepanovic et al., 2000).

The effects of honeys on biofilm formation were evaluated by means of the crystal violet static biofilm formation assay in microtiter plates. Bacteria were initially grown in tryptic soy broth (TSB, Oxoid, Basingstoke, United Kingdom) supplemented with 1% glucose. After 18 h, stationary phase cultures were harvested by centrifugation and adjusted to an OD_650_ of 0.1. To determine whether honey prevented biofilm formation, biofilms were established in microplates, using TSB supplemented with honey (2–22%, in 2% increments), by inoculating each well with 200 μL of broth culture. Plates were incubated aerobically at 37°C for 24 h. To estimate biomass, unattached cells were gently aspirated and discarded, and adherent cells were washed twice with phosphate buffer saline (PBS) and stained with crystal violet [0.25% (w/v)] for 10 min. They were subsequently washes twice with PBS and cell-bound crystal violet was resolubilized with ethanol. Absorbance was measured at 690 nm using a Multiscan Ascent (Thermo Scientific, Waltham, MA, United States).

To determine the effect of the honeys on preformed biofilms, 24-h biofilms in microtiter plates were washed twice with PBS, then 200 μL aliquots of honey dissolved in TSB (2–22%, in 2% increments) were added to each well. The plates were incubated at 37°C for a further 24 h, then they were stained with crystal violet and biofilms were assessed as described previously (Stepanovic et al., 2000; Lu et al., 2014). The *S. epidermidis* ATCC35984 has been included as a biofilm-producing reference strain.

### Transmission Electron Microscopy

The honeys’ effects on bacterial ultrastructure were studied by transmission electron microscopy (TEM) analysis conducted in a Gram-positive (*Staphylococcus aureus*) and a Gram-negative (*Pseudomonas aeruginosa*) organism.

Overnight cultures in BHI broth were diluted to obtain an OD_675_ of 0.1, approximately corresponding to 1 × 10^8^ cfu/mL. Then, 1 mL of the culture was mixed with 20 mL of brain heart infusion (BHI) agar (Oxoid) in a Petri dish. Before solidification of the medium, a well (10 mm in diameter) was created in the middle of the inoculated plate and a 100-μl aliquot of honey was placed inside. The plates were incubated overnight at 37°C. Electron microscopy was performed on agar slices taken immediately outside the zone of inhibition and from peripheral areas of the plate (positive control). The specimens were fixed in 2.5% glutaraldehyde and post-fixed in Os0_4_ in cacodylate buffer, dehydrated with alcohol, and embedded in araldite resin. Ultrathin sections were observed with CM10 electron microscope (Philips Industries, Eindhoven, Netherlands).

### Statistical Analyses

For physicochemical parameters, chemical composition, total antioxidant capacity, MDA assays and antibiofilm activity, statistical analyses were performed using IBM SPSS Statistics for Windows, version 20.0. A one-way ANOVA was used to determine significant differences amongst honey samples with a Bonferroni correction for multiple sample comparison. In all cases a *p*-value <0.05 was considered as statistically significant. The samples were analyzed in triplicate and on two different occasions. The four honeys were tested simultaneously. Results were expressed as mean ± standard deviations (SD).

## Results

### Physicochemical Parameters, Chemical Composition and Total Antioxidant Capacity

The physicochemical parameters, chemical composition and total antioxidant capacity of the four honey types are shown in **Table 1**.

**Table 1:** Physicochemical parameters, bioactive compounds and total antioxidant capacity in the four honey types in study.

Parameters	Manuka honey	African honey	*A. mellifera* honey^∗^	*M. beecheii* honey^∗^
**Physicochemical parameters**				
Color (mm Pfund)	62.11 ± 3.02^a^	121.62 ± 12.86^b^	37.35 ± 6.52^c^	41.65 ± 7.68^c^
Moisture (%)	10.22 ± 1.53^a^	12.26 ± 1.61^b^	16.74 ± 0.38^c^	28.62 ± 3.25^c^
pH	4.51 ± 0.81^a^	4.83 ± 0.21^a^	4.76 ± 0.41^a^	3.20 ± 0.21^b^
Free acidity (meq/Kg of honey)	21.63 ± 5.73^a^	23.01 ± 4.16^a^	32.65 ± 4.85^b^	41.52 ± 8.19^c^
HMF (mg/kg of honey)	19.42 ± 2.32^a^	38.87 ± 1.32^b^	16.54 ± 3.12^c^	9.23 ± 1.32^d^
Diastase index (°Gothe)	19.03 ± 213^a^	14.32 ± 2.20^b^	13.75 ± 1.52^b^	1.30 ± 0.12^c^
Electrical conductivity (mS/cm)	0.28 ± 0.026^a^	0.48 ± 0.03^b^	0.33 ± 0.02^a^	0.58 ± 0.14^c^
Ashes (%)	0.16 ± 0.01^a^	0.27 ± 0.02^b^	0.18 ± 0.04^a^	0.46 ± 0.03^c^
**Bioactive compounds**				
Total phenolic content (TPC) (mg GAE/100 g of honey)	43.16 ± 6.29^a^	126.84 ± 9.53^b^	54.30 ± 7.19^c^	94.39 ± 14.55^d^
Total flavonoid content (TFC) (mg CE/100 g of honey)	3.82 ± 0.82^a^	6.73 ± 0.70^b^	2.68 ± 0.38^c^	4.19 ± 0.37^d^
Total carotenoids content (TCC) (mg *β*carotE/kg of honey)	4.63 ± 0.46^a^	5.21 ± 0.84^b^	4.78 ± 0.34^a^	6.24 ± 0.29^c^
Vitamin C content (VitC) (mg/100 g of honey)	2.84 ± 0.28^a^	2.73 ± 0.08^a^	4.55 ± 0.87^b^	8.84 ± 0.84^c^
Total free amino acids content (mg LE/100 g of honey)	185.49 ± 8.52^a^	61.19 ± 9.31^b^	99.15 ± 12.04^c^	119.69 ± 13.95^d^
Total protein content (mg BSA/ g of honey)	2.62 ± 0.32^a^	1.35 ± 0.16^b^	1.81 ± 0.22^b^	2.71 ± 0.26^a^
Folic acid content (μg folic acid/ 100 g of honey)	0.61 ± 0.06^a^	5.46 ± 0.42^b^	8.34 ± 0.15^c^	7.37 ± 1.19^d^
**Total antioxidant capacity (TAC)**				
FRAP (*μ*mol TE/100g of honey)	126.83 ± 7.43^a^	192.92 ± 10.63^b^	159.70 ± 17.28^c^	175.82 ± 10.83^d^
DPPH (*μ*mol TE/100 g of honey)	44.63 ± 3.18^a^	64.10 ± 1.99^b^	31.06 ± 2.19^c^	42.23 ± 1.66^a^


*Sample was analyzed in triplicate and data are presented as means ± standard deviation. The mean values within a row with different letters are significantly different for p < 0.05. ^∗^Values previously reported by our group in these honey samples (Alvarez-Suarez et al., 2018).*

According to the color analysis Manuka honey was classified as light amber honey (Pfund values between 51 and 85 mm), while African honey was classified as dark honey (Pfund > 114 mm). This is also, in contrast to the *A. mellifera* and *M. beecheii* honeys that were previously classified as extra light amber honeys (Alvarez-Suarez et al., 2018). The moisture values were within the recommended values for both honey types: not exceeding 20% for *A. mellifera* and 30% for *M. beecheii* honey. The pH values in the *A. mellifera* honey samples (Manuka, African and Cuban) showed no significant differences between the three honey types. However, when compared with the *M. beecheii* honey, the pH values significantly differed between both bee species: *M. beecheii* honey had lower pH values than *A. mellifera* honey (*p < 0.05*). Similarly to pH, free acidity was higher in *M. beecheii* honey (*p < 0.05*) than in *A. mellifera* honey, confirming the acidic character of *M. beecheii* honey as compared to *A. mellifera* honey. In all honey samples the ash content and electrical conductivity values did not exceed the recommended limits (≤0.6% and <8 Schade units, respectively), suggesting that the samples have a nectar origin. Regarding the freshness indicators of honey (HMF and Diastase index), all honey samples were within the recommended limits for both indicators.

The total content of bioactive compounds differed significantly (*p < 0.05*) between the four honey types (**Table 1**). African honey had the highest values (*p < 0.05*) of TPC and TFC, compared to the rest of the honeys, whilst *M. beecheii* honey had the highest values of TCC and vitamin C content (*p < 0.05*). Manuka honey was the honey with the highest free amino acids content (*p < 0.05*), whilst the *M. beecheii* and *A. mellifera* honeys had the highest values of total protein and folic acid content, respectively. A similar behavior was found in the total antioxidant capacity results, with a marked difference in the FRAP and DPPH values between the honey types, were African honey showed the highest values of total antioxidant capacity (*p < 0.05*) following by *M. beecheii* honey.

### MAD Assessment

The MAD of the four honey types are summarized, with statistical analysis, in **Table 2**. *M. beecheii* honey, previously found to display greater antimicrobial activity than *A. mellifera* honey (Alvarez-Suarez et al., 2018), exhibited a superior activity (MAD range, 1–10%) also compared to Manuka (MAD range, 8- > 20%) and African (MAD range, 6- > 20%) honeys (*p < 0.05*). A particular difference was recorded against *C. albicans* (MAD of *M. beecheii* honey, 3%, vs. MAD of the other honeys, > 20%) (*p < 0.05*). However, *M. beecheii* honey was less active against *Streptococcus* species (MAD range, 4–10%) than against the other species tested (MAD range, 1–3%) (*p < 0.05*). This Cuban honey was also the most effective in preventing *Proteus mirabilis* swarming (MAD, 2%), however, the result wasn’t statistically significant.

**Table 2:** *In vitro* antimicrobial activity of Manuka, African, *A. mellifera*, and *M. beecheii* honeys against 52 clinical isolates^∗^.

Strain	Manuka	African	*A. mellifera*	*M. beecheii*
*S. aureus* (*n* = 5)	8.0 ± 0.3^a^	6.0 ± 0.1^b^	16.0 ± 0.8^c^	2.0 ± 0.1^d^
*S. epidermidis* (*n* = 5)	9.0 ± 0.8^a^	7.0 ± 0.4^b^	14.0 ± 0.6^c^	1.0 ± 0.02^d^
*S. pneumoniae*	10.0 ± 0.6^a^	10.0 ± 0.7^a^	11.0 ± 0.4^a^	4.0 ± 0.4^b^
*S. pyogenes* (*n* = 5)	12.0 ± 0.4^a^	16.0 ± 0.2^b^	15.0 ± 0.2^b^	6.0 ± 0.2^c^
*S. agalactiae*	14.0 ± 0.8^a^	>20.0 ± 0.5^b^	19.0 ± 0.2^b^	10.0 ± 0.6^c^
*S. mitis*	11.0 ± 0.2^a^	14.0 ± 0.3^a^	14.0 ± 0.7^a^	7.0 ± 0.3^b^
*S. oralis*	10.0 ± 0.6^a^	12.0 ± 0.3^a^	11.0 ± 0.2^a^	6.0 ± 0.2^c^
*S. anginosus*	11.0 ± 0.4^a^	14.0 ± 0.6^a^	14.0 ± 0.6^a^	8.0 ± 0.4^b^
*S. parasanguinis*	12.0 ± 0.6^a^	14.0 ± 0.2^a,b^	15.0 ± 0.4^b^	8.0 ± 0.2^c^
*S. salivarius*	12.0 ± 0.1^a^	15.0 ± 0.6^b^	14.0 ± 0.5^b^	7.0 ± 0.1^c^
*S. gordonii*	11.0 ± 0.4^a^	13.0 ± 0.7^a^	11.0 ± 0.6^a^	6.0 ± 0.2^b^
*E. faecalis* (*n* = 5)	11.0 ± 0.6^a^	14.0 ± 0.4^a^	>20.0 ± 0.4^b^	3.0 ± 0.06^c^
*E. faecium* (*n* = 5)	10.0 ± 0.7^a^	9.0 ± 0.2^a^	18.0 ± 0.3^b^	2.0 ± 0.02^c^
*L. monocytogenes*	9.0 ± 0.1^a^	9.0 ± 0.6^a^	17.0 ± 0.3^b^	3.0 ± 0.01^c^
*E. cloacae*	12.0 ± 0.7^a^	9.0 ± 0.4^b^	15.0 ± 0.8^c^	2.0 ± 0.02^d^
*C. freundii*	12.0 ± 0.6^a^	9.0 ± 0.6^b^	14.0 ± 0.6^c^	2.0 ± 0.01^d^
*S. fyris*	14.0 ± 0.4^a^	10.0 ± 0.4^a^	20.0 ± 0.7^b^	3.0 ± 0.03^c^
*S. marcescens*	19.0 ± 0.3^a^	9.0 ± 0.3^b^	16.0 ± 0.8^c^	2.0 ± 0.01^d^
*A. baumannii*	11.0 ± 0.2^a^	8.0 ± 0.5^b^	19.0 ± 0.2^c^	3.0 ± 0.04^d^
*K. pneumoniae*	14.0 ± 0.5^a^	11.0 ± 0.4^b^	17.0 ± 0.4^c^	2.0 ± 0.06^d^
*P. aeruginosa* (*n* = 5)	14.0 ± 0.4^a^	9.0 ± 0.1^b^	11.0 ± 0.8^c^	2.0 ± 0.02^d^
*E. coli* 23 (*n* = 5)	11.0 ± 0.9^a,b^	10.0 ± 0.5^a^	13.0 ± 0.1^b^	3.0 ± 0.01^c^
*P. mirabilis*	14.0 ± 0.6^a^	7.0 ± 0.2^b^	13.0 ± 0.3^a^	2.0 ± 0.04^c^
	4.0 ± 0.1^a,c∗∗^	6.0 ± 0.3^a∗∗^	9.0 ± 0.5^b∗∗^	2.0 ± 0.1^c∗∗^
*C. albicans*	>20.0 ± 0.6^a^	20.0 ± 0.8^a^	>20.0 ± 0.3^a^	3.0 ± 0.2^b^


*Sample was analyzed in triplicate in two different occasions and data are presented as means ± standard deviation. Mean values within a row with different letters (a–d) are significantly different for *p < 0.05*. ^∗^We have already recently reported data on *M. beecheii* and *A. mellifera* honeys against this strains collection (Alvarez-Suarez et al., 2018). Some of the data are reported here again to make comparison with other honeys easier. ^∗∗^This value indicates the honey dilution which is able to prevent *P.mirabilis* swarming.*

### Effects of the Four Honeys on Microbial Biofilm Formation

One strain for each species was assessed for its biofilm-forming ability after 24 h; 5/23 clinical isolates (*S. aureus* 13, *Staphylococcus epidermidis* 35, *Streptococcus pyogenes* 12, *Streptococcus gordonii* 143, and *P. aeruginosa* 24) turned out to be strong biofilm producers and were chosen to evaluate the honeys’ effects on biofilm formation. In addition, *S. epidermidis* ATCC35984 was used as control (**Supplementary Figure S1**).

**FIGURE 1:**
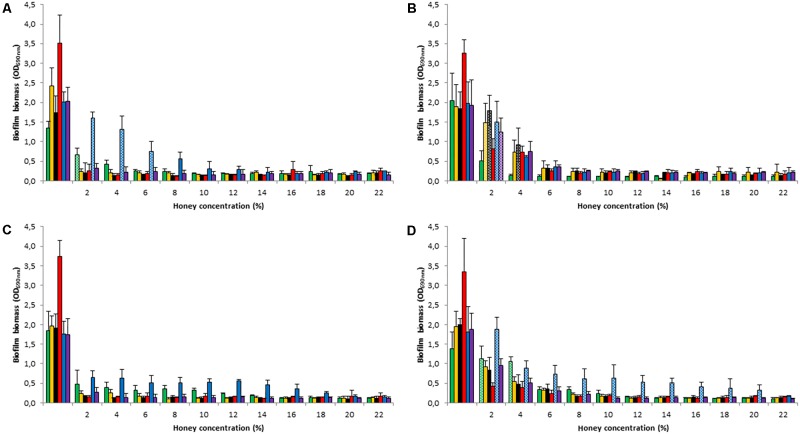
Honey’s effect on biofilm formation. Inhibition of biofilm development using Manuka **(A)**, *M. beecheii* **(B)**, *A. mellifera* **(C)**, and African **(D)** honeys. Biofilms of *S. aureus* (green), *S. epidermidis* (orange), *S. pyogenes* (black), *S. gordonii* (red), *P. aeruginosa* (blue), and *S. epidermidis* ATCC35984 (violet) were grown for 24 h in TSB supplemented with honey (2–22%). Biofilm formation is expressed as absorbance at 690 nm. Error bars represent ± standard deviation (SD). Non-significant values (*p* > 0.05), compared to the control, are represented by dotted histograms.

When biofilms were established in the presence of honey concentrations of 2–22%, a significant reduction in biomass was consistently observed at concentrations ≥ 8% (*p < 0.05*) (**Figure 1**). Remarkably, *M. beecheii* honey was the most effective in preventing *S. epidermidis*, *S. pyogenes*, and *S. gordonii* biofilm formation and was already active at 2% (*p < 0.05*). The inhibiting effect of Manuka honey, at low concentrations (2 – 6%), on the formation of *S. aureus* biofilm was greater than that of the other honeys (*p < 0.05*). Overall, the African honey was the least effective and *P. aeruginosa* was the species least susceptible to the honeys’ inhibition of biofilm production, however the results weren’t statistically significant.

### Effects of Honeys on Established Biofilms

**FIGURE 2:**
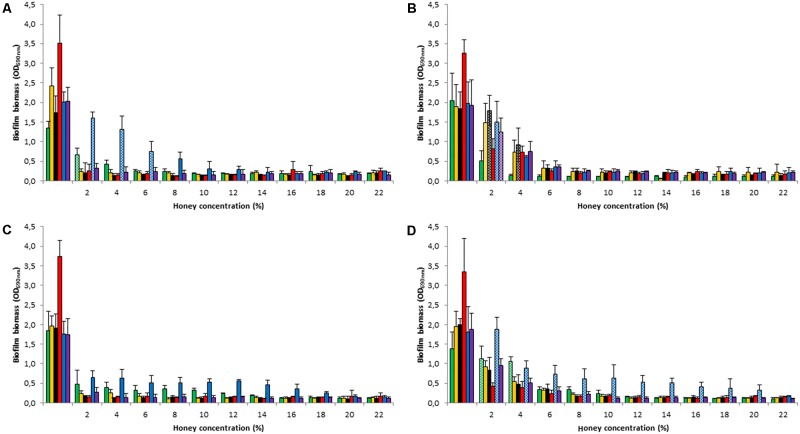
Honey’s effect on established biofilms. Reduction in biofilm mass following treatment by Manuka **(A)**, *M. beecheii* **(B)**, *A. mellifera* **(C)**, and African **(D)** honeys (2–22%) on 24-h established biofilms of *S. aureus* (green), *S. epidermidis* (orange), *S. pyogenes* (black), *S. gordonii* (red), *P. aeruginosa* (blue), and *S. epidermidis* ATCC35984 (violet). Biofilm formation is expressed as absorbance at 690 nm. Error bars represent ± standard deviation (SD). Non-significant values (*p* > 0.05), compared to the control, are represented by dotted histograms.

We also assessed the ability of the four honey types to remove established biofilms produced by the five test strains and the *S. epidermidis* ATCC35984. The results were consistent with those described above for biofilm formation inhibition (**Figure 2**). All types of honey induced a pronounced reduction of established biofilms, at concentrations ≥8% (*p < 0.05*), but without leading to their complete eradication. Interestingly, *M. beecheii* honey, already at a concentration of 2%, produced a reduction in biofilm mass of over 70% in *S. epidermidis*, *S. pyogenes*, and *S. gordonii*, but, as well as *A. mellifera* honey, had low activity against *P. aeruginosa* (*p < 0.05*).

### Ultrastructural Studies

The effect of *M. beecheii* honey, showing the greatest antimicrobial activity, on bacterial ultrastructure was investigated in *S. aureus* 13 and *P. aeruginosa* 24.

**FIGURE 3:**
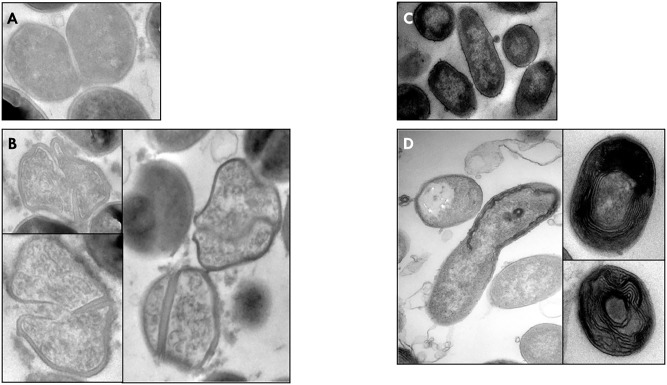
Electron microscopic analysis. The *M. beecheii* honey’s effect on *S. aureus* **(B)**, and *P. aeruginosa* **(D)** cellular structure. Untreated cells of *S. aureus* **(A)** and *P. aeruginosa* **(C)**. Original magnification, X44,000.

Compared to untreated cells, honey-treated *S. aureus* 13 cells displayed wall thickening, non-homogeneous cytoplasm, and unpreserved morphology. However, the most significant changes were observed during cell division, when daughter cells did not fully separate, with the septum being thickened and often not completely formed (**Figures 3A,B**).

In *P. aeruginosa* 24, honey-induced morphological modifications were even more distinct, but did not involve cell division. The presence of cellular debris in TEM images indicated cells lysis. Interestingly, cross-sections of honey-treated bacteria exhibited multilayered concentric structures (**Figures 3C,D**).

## Discussion

The worldwide emerging antibiotic resistance and the increasing unavailability of drugs active against multidrug resistant bacteria necessitates the search for alternative antimicrobial strategies. The need for new therapeutic approaches is at the base of the renewed interest in a number of natural products showing antimicrobial activity, in particular honey for potential topical use (Maddocks and Jenkins, 2013; Cooper, 2016).

A large number of studies of New Zealand/Australian Manuka honey have shown that (i) it has broad spectrum activity against pathogenic bacteria (Willix et al., 1992; Karayil et al., 1998; French et al., 2005); that (ii) such activity is independent of susceptibility or resistance to common antibiotics (Willix et al., 1992; Karayil et al., 1998; Cooper et al., 2002); and that (iii) honey-susceptible organisms — unlike what easily happens with systemic antibiotics and other topical antimicrobials — are apparently unable to turn honey-resistant (Blair et al., 2009; Cooper et al., 2010; Maddocks and Jenkins, 2013). Conversely, very limited data are available on the antimicrobial activity of other honeys, and this study was aimed at reducing this gap by comparing Manuka with two recently investigated Cuban honeys (Alvarez-Suarez et al., 2018) and an African honey.

The chemical composition of honey is important information that can be used to justify its biological properties. The four honey types here analyzed showed to be an important natural source of bioactive compounds such as polyphenols, flavonoids, vitamin C and folates. Similarly, all honey showed an important total antioxidant capacity that has been previously correlated with the content of bio-compounds and their biological properties (Alvarez-Suarez et al., 2010b, 2018).

Our results indicate that Cuban *M. beecheii* honey demonstrates potent inhibitory activity — greater than that of other honeys including Manuka — against all clinical isolates tested, that included representatives of Gram-positive, Gram-negative, and *C. albicans*. Overall, the honeys exert their antimicrobial effect regardless of the microorganism, suggesting a non-specific action. On the other hand, it is well-established that differences in chemical composition, largely due to different botanical origin, may account for substantial inter-honey variability (Voidarou et al., 2011). Very recently, we showed that different physicochemical parameters are responsible for the higher antimicrobial activity of *M. beecheii* honey compared to *A. mellifera* honey (Alvarez-Suarez et al., 2018).

In particular, honey is regarded as potentially effective in the management of biofilm-associated infections: previous studies have demonstrated that honey may both hinder biofilm formation and reduce preformed biofilms (Alandejani et al., 2008; Jervis-Bardy et al., 2011; Kilty et al., 2011; Maddocks et al., 2012, 2013; Nassar et al., 2012; Majtan et al., 2014; García-Tenesaca et al., 2018). Here we showed that four honeys inhibit biofilm production by *S. aureus*, *S. epidermidis*, *S. pyogenes*, *S. gordonii*, and *P. aeruginosa* strains, and reduce preformed biomass without complete biofilm eradication. Overall, *M. beecheii* honey showed the strongest antibiofilm effect, and *P. aeruginosa* proved to be the species least susceptible to honey effect on biofilm inhibition.

The effect of *M. beecheii* honey on cellular ultrastructure was investigated by TEM in *S. aureus* and *P. aeruginosa*. Strong thickening of the septum with alteration of bacterial morphology was observed in *S. aureus*. Previous studies indicated that autolysines are involved in the failure of *S. aureus* cells to progress normally through cell cycle to form thickened septa during division without cell separation (Henriques et al., 2010; Jenkins et al., 2011, 2014). It is reasonable to assume that *M. beecheii* and Manuka honeys have a similar mechanism of action involving the cell division machinery. Particularly marked structural changes were seen in honey-treated *P. aeruginosa* cells: in addition to a previously described loss of structural integrity (Henriques et al., 2011), multilayered concentric structures were observed.

Among the physicochemical parameters of honey, the acidity and the osmolarity represent the principal factors responsible for the antimicrobial activity of honey. However, there are other factors that are closely related to the antimicrobial capacity of honey such as the hydrogen peroxide content, and other non-peroxide components such as methylglyoxal, the antimicrobial peptide bee defensin-1, polyphenols and other compounds from the bees (Scepankova et al., 2017).

In this study, *M. beecheii* honey showed a more pronounced acid character (pH and free acidy values) compared to Manuka, African, and *A. mellifera* honeys; this result, as well as the high TPC, could explain the greatest *M. beecheii* honey antimicrobial activity.

## Conclusion

Cuban *M. beecheii* honey displayed the highest antimicrobial activity, greater than that of the well-established and medically available Manuka, and should be regarded as an excellent candidate for further studies aimed at providing valuable insights into the topical issue of the treatment of chronic wound infections due to pathogens that do not respond to antibiotic therapy. It is noteworthy that *M. beecheii* honey also showed a noticeable antioxidant capacity known for promoting the healing of wounds. Further experiments will be required to clarify which honey compounds actually play a role in antimicrobial activity.

## Author Contributions

EG, AB, and MB designed the study. EG and AB collected and analyzed the data, and wrote the paper together with JA-S. GM, SS, EM, MM, and SF did the laboratory work in order to investigate the honey’ antimicrobial activity. AP studied the honeys’ effects on bacterial ultrastructure by transmission electron microscopy (TEM) analysis. JA-S, FG, LM, and MG characterized the honeys for physicochemical parameters, chemical composition and total antioxidant capacity.

## Conflict of Interest Statement

The authors declare that the research was conducted in the absence of any commercial or financial relationships that could be construed as a potential conflict of interest.

## Supplementary Material

FIGURE S1 One strain for each species was assessed for its biofilm-forming ability after 24 h. OD_690_ values above the red line indicate strong biofilm producer strains. The cut-off was deduced as suggested by Stepanovic et al. (2000).
