# Supplementary figures and images for: Comparison of the Antimicrobial Activities of Four Honeys From Three Countries (New Zealand, Cuba, and Kenya)
Source: Front Microbiol. 2018 Jun 25;9:1378. doi: 10.3389/fmicb.2018.01378 (PMC6026664; doi:10.3389/fmicb.2018.01378)

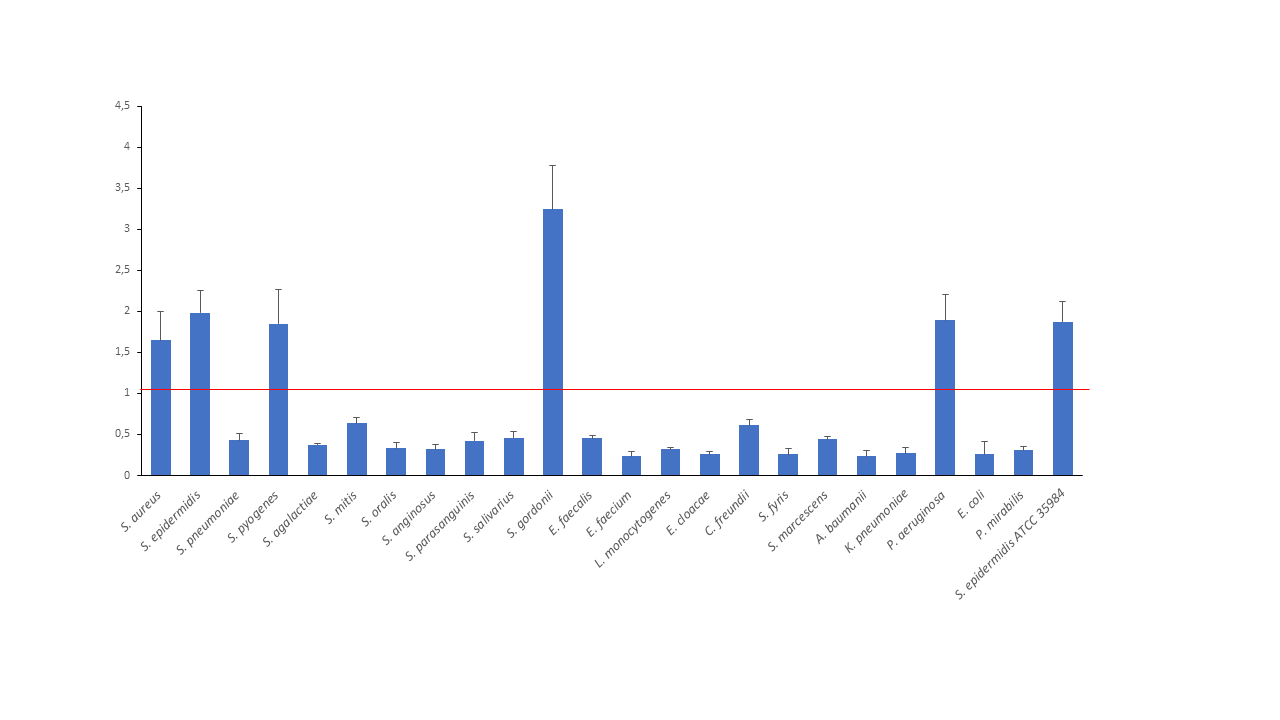

Supplement: FIGURE S1 — One strain for each species was assessed for its biofilm-forming ability after 24 h. OD690 values above the red line indicate strong biofilm producer strains. The cut-off was deduced as suggested by Stepanovic et al. (2000). [file Image_1.TIF]
